# Clozapine: Current perspective

**DOI:** 10.4103/0019-5545.37668

**Published:** 2007

**Authors:** Ram K. Solanki, Paramjeet Singh, Mukesh K. Swami

**Affiliations:** Psychiatry Center, Department of Psychiatry, SMS Medical College, Jaipur, India

**Keywords:** Clozapine, resistant schizophrenia, SGA

## Abstract

The author conducted a review of studies that compared the efficacy, tolerability and indication for the use of clozapine in current perspectives for the treatment of resistant schizophrenia/ partial responders.

## INTRODUCTION

In the early 1960s, German psychiatrists working with G. Stille at Wander Pharmaceuticals in Bern, Switzerland, worked to refute the concept that EPS and antipsychotic efficacy were linked.[1] Their work led to the introduction of clozapine, an antipsychotic with no EPS or minimally associated EPS.[2,3] Clinical confirmation of this profile for clozapine was provided in open studies by Austrian[4] and German[5] investigators in 1966 and later by Swiss researchers[6] in a double-blind study in 1971. Clozapine was briefly marketed and quickly withdrawn.[3]

However, enthusiasm for the drug was maintained by a small cadre of clinical investigators including G. Honigfeld at Sandoz, who observed that clozapine, was remarkably effective in treatment-resistant patients. This led to a landmark double-blind study of clozapine in a well-defined group of treatment-resistant patients whose blood cell counts were closely monitored during treatment[7] and ultimately to its introduction to the US market in 1990. Clozapine was approved by the FDA in 1990.[1,7] Clozapine was also approved for reducing the risk of suicide in schizophrenic or schizoaffective patients judged to be at chronic risk for suicidal behavior in December 2002.

### Mode of action

The mechanisms of action which account for the effectiveness of clozapine as a pharmacotherapy for the treatment of neuroleptic non-responders and neuroleptic intolerant schizophrenic subjects remain elusive. It is characterized by generally lower affinities for D2 receptors and relatively greater affinities for serotonin (5-hydroxytryptamine) 5-HT2A receptors in particular, but also for noradrenergic receptors (α1 and α2), muscarinic acetylcholine receptors, histamine and other dopamine (DA) subtype receptors.

Tauscher *et al*,[8] used Positron emission tomography with the radioligands [^11^C] SCH23390 and [^11^C]raclopride to investigate D_1_ and D_2_ receptor occupancy *in vivo* in 25 schizophrenic patients receiving atypical antipsychotic treatment with clozapine, olanzapine, quetiapine or risperidone. They concluded that among the atypical antipsychotics, clozapine appeared to have a simultaneous and equivalent occupancy of dopamine D_1_ and D_2_ receptors. Whether its effect on D_1_ receptors represents agonism or antagonism is not yet clear. This issue is still unresolved in the preclinical arena. The distinctive effect on D_1_/D_2_ receptors may be responsible for clozapine's unique effectiveness in patients with schizophrenia refractory to other typical and atypical antipsychotics.

### Efficacy

Meta analysis of controlled trials involving patients who had treatment resistant schizophrenia[9–12] and a systemic review[13] showed that clozapine is as effective or is more effective than the other second generation antipsychotics for treating treatment resistant schizophrenia.

In an open trial by Agarwal *et al.*,[14] drug-resistant schizophrenics were treated with clozapine for sixteen weeks. The patients were rated on BPRS, PANSS, Side effects scale and Global Impression Scale at weeks 0, 9 and 16. A battery of base-line investigations was done and hemogram was repeated at weekly intervals. Of the total 29 patients included, 25 completed the trial. The patients showed significant improvement on both BPRS and PANSS, though the improvement was more in initial weeks than between 9 and 16 weeks. The most common side effects observed were sedation, hypersalivation and tachycardia.

Moncrieff[12] compared clozapine with conventional antipsychotics and challenged previous results, criticizing methodological bias in the clinical trials. He found that recent large-scale studies have not found a substantial advantage for clozapine, especially in terms of a clinically relevant effect. Meta-regression showed that shorter study duration, financial support from a drug company and higher baseline symptom score consistently predicted greater advantage of clozapine. He concluded that the superiority of clozapine over conventional antipsychotic drugs has not been consistently and conclusively demonstrated. There is substantial variation between results of different studies, which appears to be accounted for by study duration and funding and level of initial symptoms.Clozapine's greatest advantage relative to standard medication may be seen in patients with very high levels of initial symptoms.

Several authors have questioned whether clozapine should be indicated as a first-line treatment for early psychosis.[15–17] The risk-benefit ratio has been reappraised[18] in view of the lower rates of relapse, hospitalization, extrapyramidal symptoms and suicidality and the improvements in negative symptoms, cognition and social functioning associated with clozapine.

Woerner *et al.*, reported the interesting observation that clozapine, in comparison with fluphenazine, was similarly efficacious for new-onset patients with schizophrenia or schizoaffective disorder.[19] It is frequently questioned, why is clozapine, compared with typical antipsychotics, dramatically more efficacious for treatment-resistant psychosis but merely similarly efficacious for new-onset schizophrenia? To provide an explanation it is hypothesized[20] that patients taking long-term neuroleptic medication experience upregulation of dopamine D_2_ receptors in their brains. Typical antipsychotics may cause the upregulation because they dissociate slowly from the D_2_ receptor[21] and the neurons respond by trying to overcome the chronic blockade. In contrast, clozapine, which is a fast dissociator,[21] may allow endogenous dopamine to activate the receptor enough to avoid the problem of upregulation. In addition to dopaminergic upregulation in the nigrostriatal tracts (thought to underlie the pathophysiology of tardive dyskinesia), many investigators have suggested that dopaminergic upregulation may occur in mesolimbic or mesocortical tracts, leading to a worsening of psychosis beyond the original level. This phenomenon has been called “tardive psychosis” or “supersensitivity psychosis”.[22] Because increasing the dose of the neuroleptic eventually will make the problem worse, tardive psychosis may be a major form of treatment-resistant psychosis. Clozapine, in comparison with typical antipsychotics, may be more efficacious for treatment-resistant psychosis because it allows the reversal of the dopaminergic upregulation in the mesolimbic or mesocortical tracts, leading to an improvement in tardive psychosis. On the other hand, clozapine, in comparison with typical antipsychotics, may not be more efficacious for new-onset schizophrenia because these patients have not been medicated previously and do not have dopaminergic upregulation that can be reversed.[20]

Pragmatic trials, supposed to provide more complete information for physician in clinical practice,[23] support the effectiveness of clozapine over newer atypical antipsychotics. Evoy *et al.*,[24] in a phase 2 trial in the CATIE study,[25] studied 99 patients who had not responded to atypical antipsychotics in previous phase.[26] Patients are randomly assigned to open label clozapine (n=49) or to blind treatment with another SGA (olanzapine, n=19; quitiapine, n=15; risperidone, n=16). When compared with other SGA's, clozapine had the greatest reduction in the PANSS total score and lowest discontinuation rates; that is the use of clozapine proved to be more effective than switching to another second generation antipsychotic in patients who previously had not responded to another second generation antipsychotics.

In another pragmatic trial Lewis *et al*,[27] studied 137 patients who had schizophrenia and who had showed a poor response to 2 or more antipsychotics.. The patients were assigned randomly to receive clozapine (n=67) or a second generation antipsychotics (risperidone, olanzapine, quetiapine, amisulpride). At the end of 1 year patients who received clozapine showed a reduction in psychopathology and improved quality of life.

In a Meta analysis comparing clozapine with a second generation antipsychotic, Tuunainen *et al*,[11] found a trend for clozapine to be more effective in improving positive symptoms but not negative symptoms; no difference were seen in other outcome variables, such as relapse rates or global improvement. There was a trend for a second generation antipsychotics to be more effective in improving cognition.

In a Double-Blind Comparative Study,[28] including 273 patients with poor previous treatment response, Azorin *et al.*, found that the magnitude of improvement in mean BPRS and CGI scores from baseline to end of the study was significantly greater in the clozapine group than in the risperidone group. Statistically significant differences in favor of clozapine were also seen for most of the secondary efficacy measures (Positive and Negative Syndrome Scale, Calgary Depression Scale, Psychotic Depression Scale and Psychotic Anxiety Scale). The adverse event profile was similar for both treatment groups, with a lower risk of extrapyramidal symptoms in the clozapine group.

Clozapine has shown consistent clinical benefit in schizophrenic patient with persistent aggressive and violent behavior.[29,30] Whether this is due to a sedative effect, a specific antiaggressive action or just reflects an overall improvement in psychosis is unknown.

McGurk *et al.*,[31] investigated the effects of clozapine and risperidone on spatial working memory in patients with schizophrenia. Spatial working memory performance was evaluated at baseline and after 17 and 29 weeks in 97 patients with schizophrenia participating in a multisite trial. They found that compared with baseline performance while receiving conventional antipsychotic medication, risperidone improved and clozapine worsened, spatial working memory performance. The differential effects of these medications on spatial working memory may be due to the anticholinergic effects of clozapine and prefrontal dopamine-enhancing effects of risperidone.

Bilder *et al.*,[32] examined the effects of clozapine, olanzapine, risperidone and haloperidol on 16 measures of neurocognitive functioning in a double-blind, 14-week trial involving 101 patients. A global score was computed along with scores in four neurocognitive domains: memory, attention, motor function and general executive and perceptual organization. They found that global neurocognitive function improved with olanzapine and risperidone treatment and these improvements were superior to those seen with haloperidol. Patients treated with olanzapine exhibited improvement in the general and attention domains but not more than that observed with other treatments. Patients treated with risperidone exhibited improvement in memory that was superior to that of both clozapine and haloperidol. Clozapine yielded improvement in motor function but not more than in other groups.

Purdon *et al.*,[33] examined Neuropsychological change after 6 weeks of clozapine treatment in 18 treatment-refractory patients to test anticipated domain-specific cognitive improvements. The results showed that the comprehensive neuropsychological test battery was sensitive to general cognitive changes with clozapine and supported the hypothesized domain-specific gains on tests of motor and mental speed, visual spatial manipulation and new verbal learning. Novel gains were also apparent on tests of new learning with nonverbal material.

### Augmentation studies

It is estimated that approximately 30% of patients treated with clozapine do not respond adequately, remaining with persistent psychotic symptoms despite taking adequate treatment for sufficient period. Such patients are called “partial responders to clozapine,” “clozapine resistant,” or even “super refractory”.[34] Despite lack of evidence augmentation strategies are used in such patients. Various antipsychotics have been used to augment clozapine: risperidone,[35,36] amisulpride,[37,38] aripiprazole,[39] haloperidol,[40] loxapine,[41] olanzapine,[42] pimozide[43] and ziprasidone.[44] The benefits of such augmentation strategies remain inconclusive because they were tested in case series or case reports, which have a low strength of evidence as compared with controlled trials.[45,46]

In a study conducted at psychiatry center, S.M.S. medical college, Jaipur, by Dr. R.K. Solanki and Dr. Paramjeet Singh, 100 diagnosed cases of schizophrenia on clozapine maintenance (not showing adequate response) were augmented with risperidone (2-4 mg/d). Patients showed significant improvement after 4 week on CGI, BPRS and PANSS scales.

### Other therapeutic uses

Lieberman *et al.*,[47] reviewed eight published studies that describe clozapine's effects on tardive dyskinesia and examined the outcome of 30 patients with tardive dyskinesia treated withclozapine for up to 36 months. They found that tardive dyskinesia response toclozapine is variable but that approximately 43% of cases, particularly those with dystonic features, improved after clozapine treatment.

In international suicide prevention trial[48] including 980 patients from 67 medical centers in 11 countries (assigned randomly to either treatment with clozapine or olanzapine) it is concluded that the rate of suicidal behavior or suicide attempts are significantly lower in patient taking clozapine than in those taking olanzapine, although the rates of deaths from suicide were not significantly different between groups.

Turetz *et al.*,[49] treated 11 neuroleptic-resistant children (< 13 years) with schizophrenia in an open trial. The mean clozapine dosage was 227.3 mg. There was an overall statistically significant reduction in all parameters, especially positive symptoms, implying a favorable outcome. Most of the improvement occurred during the first 6 to 8 weeks. The major side-effects were somnolence and drooling (no agranulocytosis).

Green *et al.*,[50] examined efficacy of clozapine in a group of patients (n=22) with treatment-refractory bipolar disorder, manic type with psychotic features and suggested that clozapine is an effective agent for patients with treatment-refractory psychotic mania. Similar results are reported previously by Calabrese *et al*.[51]It is also suggested thatclozapine could be efficient in psychotic refractory depression.[52]

Clozapine is least likely to worsen the neurological disorder[53] so it is preferred in psychotic patient with Parkinson disease. Some neurologists even consider clozapine to be an antipsychotics of choice in these patients.[54]

### Predictors of response

A number of authors have investigated the factors associated with response to clozapine but findings are contradictory. Reviewing the evidence Chung and Remington[55] suggested that as yet there are no predictors of response to clozapine; rather there are markers. The reduction in frontal cortex metabolism seen with positron mission tomography, the reduction in caudate volume seen with MRI, the baseline severity of psychopathology and plasma clozapine level can not be considered predictors of response because of inconsistency of data.

### Side effect profile

Apart from common side effects like sedation, dizziness, syncope, tachycardia, hypotension, ECG changes, nausea and vomiting, fatigue, weight gain, constipation, anticholinergic effects and subjective muscle weakness, several rare side effects has been described during clozapine treatment. One most important is neutropenia which may lead to fatal agranulocytosis, occurs in about 1% patient. Its mechanism is unclear but may be the result of direct toxicity or an immune response.[56] Annan LJA and Andrews CD[57] investigated the results of a rechallenge in 53 patients who has previously experienced leucopenia or neutropenia during clozapine therapy. Of 53 patients who were rechallenged, 20 (38%) experienced a further blood dyscrasia. In 17 of these 20 patients (85%) the second blood dyscrasia was more severe (*P*<0.001), in 12 (60%) it lasted longer (*P*=0.0368) and in 17 (85%) it occurred more quickly on rechallenge (*P*<0.001).they concluded that after risks and benefits have been considered, rechallenge may well be justified in some patients.

In a review Wetterling suggested that Average weight gain with clozapine is 1.72 kg /month, occurring most frequently during first 6 to 12 month of treatment.[58] There have been also reports of glucose intolerance during clozapine treatment.

In a 5 year naturalistic study Henderson *et al.*,[59] examined the incidence of treatment-emergent diabetes mellitus in relation to other factors, including weight gain, lipid abnormalities, age, clozapine dose and treatment with Valproate. Mean age at the time of clozapine initiation of the 82 patients was 36.4 years. The mean baseline weight was 175.5 lb and the mean body mass index was 26.9 kg/m^2^. Thirty patients (36.6%) were diagnosed with diabetes during the 5-year follow-up. Weight gain, use of Valproate and total daily dose of clozapine were not significant risk factors for developing diabetes mellitus. Patients experienced significant weight gain that continued until approximately month 46 from initiation of clozapine. There was a nonsignificant increase in total serum cholesterol and a significant increase in serum triglycerides level.

Howes *et al.*,[60]examined the effect of clozapine on glucose control and insulin sensitivity. Glucose homeostasis was measured in nine female and 11 male patients with schizophrenia (mean age=30.5 years, SD=7.4) before clozapine treatment and after a mean of 2.5 months (SD=0.95) of clozapine treatment. They concluded that Clozapine impairs glucose control within 4 months of treatment, independent of changes in insulin sensitivity and body mass index. Recently the effect of clozapine and of haloperidol on electrical and secretory activity of pancreatic beta cells was studied: while at lower glucose concentrations, clozapine had little effect on membrane potential, at higher doses, it led to marked depolarization of the membrane potential, despite differing glucose concentrations.[61] Similarly clozapine was shown to increase basal insulin release, in contrast to conventional antipsychotics.[62]

Akathisia occurs even occasionally with clozapine.[63] Very rarely tardive dyskinesia has been reported in patient receiving clozapine.[64]Sachdev *et al.*,[65] reviewed the published case reports of clozapine-induced neuroleptic malignant syndrome (NMS) and suggested that typical NMS does occur with clozapine and that its incidence may be as common as with the classic neuroleptics. The features of clozapine-induced NMS may be somewhat different, with fewer extrapyramidal side effects and a lower rise in creatine kinase levels. Atypical antipsychotic including clozapine are sometime associated with an atypical, but potentially lethal, neuroleptic malignant-like syndrome that is marked by fever and delirium without muscle rigidity.[66]

Cohen *et al.*,[67] conducted a study to examine the arrythmogenic effects of neuroleptics. Heart rate analysis was carried out in patients with schizophrenia on standard doses of neuroleptic monotherapy - 21 were on clozapine, 18 on haloperidol and 17 on olanzapine - and in 53 healthy subjects. They found that the patients with schizophrenia on clozapine had significantly higher heart rate, lower heart rate variability and lower high-frequency and higher low-frequency components compared with patients on haloperidol or olanzapine and matched control subjects and showed autonomic dysregulation and cardiac repolarisation changes. There have been isolated reports of paradoxical hypertension with clozapine use.[68,69] Myocarditis is a rare and potentially life-threatening complication of clozapine. There are reports of reversible myocarditis during treatment with clozapine for chronic resistant schizophrenia. The patient recovered rapidly on withdrawal of clozapine and with supportive management.[70] There is also report of clozapine-induced cardiac failure.[71]

Clozapine is known to increase seizure frequency. In a review[72] seizure frequency is calculated to be 1% at a dose less than 300 mg/day, 2.7% at 300 to 599 mg and 4.4% with dose 600 mg or more daily. Valproate is recommended as the standard therapy for the treatment of clozapine-induced seizures.[73] Gabapentin is also used as an alternative.[74,75] There is also report of successful use of Topiramate for the treatment of clozapine-induced seizures.[76]

Clozapine specifically has two important risks of intestinal dysfunction: potentially severe ileus[77] and sialorrhea, which may be related to deficient pharyngeal-esophageal clearing mechanisms most noticeable during sleep.[78] Sialorrhea begins early in treatment and most evident in night. Hypersalivation has been reported[79] to occur in about 54% patient receiving clozapine.

*De novo* emergence or exacerbation of obsessive-compulsive (OC) symptoms during treatment with clozapine has been described in the literature. Lykouras *et al.*,[80] reviewed the reported cases (30 case reports) and discussed the possible pathogenetic mechanisms involved in their occurrence. He concluded that a temporal relationship between the drug and the symptoms can not be established in the cases receiving clozapine and current data are insufficient to suggest a pattern. There are some reports from India also.[81]

Toxic delirium can occur in about 3% of patient.[82]Clozapine rarely induces allergic complications.[83] There is also evidence that clozapine may be associated with fatal thromboembolism,[84] acute interstitial nephritis[85] and pancreatic.[86]

Data regarding the potential teratogenacity of clozapine are not unanimous. There are some reports of congenital malformations and other complications during pregnancy. But there is report[87] of successful use of clozapine in pregnant woman with treatment-resistant schizophrenia.

## CONCLUSION

The ten years of clinical experience of clozapine use in the dose range 100-400 mg/day as monotherapy or augmentation has been significantly safe and acceptable at large by patient subgroups. The side effect profile remained comparable to other SGA's, as evident from the unpublished data of the ongoing study at psychiatry center, SMS medical college, jaipur.

The result of the different studies indicated that clozapine exhibit superiority over typical antipsychotics in terms of both efficacy (as shown by an improvement in overall psychopathology) and safety. However the magnitude of its effect is not consistent. Efficacy data for other SGA's in the treatment of refractory schizophrenics were inconclusive.

So there is growing need to consider different treatment strategies. Whether monotherapy or adjuvant therapy for non responsive or partially responsive schizophrenics.
